# Draft Genome Sequences of *Streptococcus pneumoniae* with High-Level Resistance to Respiratory Fluoroquinolones

**DOI:** 10.1128/genomeA.00181-16

**Published:** 2016-03-31

**Authors:** Yoram Keness, Naiel Bisharat

**Affiliations:** a Microbiology Laboratories, Emek Medical Center, Afula, Israel; b Department of Medicine D, Emek Medical Center, Afula, Israel; c Bruce Rappaport Faculty of Medicine, Technion–Israel Institute of Technology, Haifa, Israel

## Abstract

*Streptococcus pneumoniae* is the leading cause of community-acquired pneumonia. Levofloxacin is a fluoroquinolone used for treatment of severe community-acquired pneumonia. Here, we describe the draft genome sequences of *S. pneumoniae* with emerging resistance to levofloxacin, resulting in failure of treatment of pneumococcal pneumonia.

## GENOME ANNOUNCEMENT

*Streptococcus pneumoniae* is the leading cause of community-acquired pneumonia (CAP). Current guidelines for empirical treatment of CAP in patients with comorbidities and recent antibiotic therapy recommend using respiratory fluoroquinolones (1). Resistance to fluoroquinolones in *S. pneumoniae* is most frequently associated with mutations in the quinolone-resistance-determining regions of *parC* and *parE* genes coding for topoisomerase IV and/or *gyrA* and *gyrB* coding for DNA gyrase (2). A single *parC* mutation represents the first mutational step conferring low-level resistance to fluoroquinolones, while a second mutational step in the *gyrA* gene confers high-level resistance (2).

Here, we describe the draft genome sequences of two *S. pneumoniae* strains, isolated from a patient suffering from pneumococcal pneumonia initially treated with a respiratory fluoroquinolone (levofloxacin) to which the strain was sensitive (strain SP224896). After 10 days of treatment with satisfactory outcome, the patient was readmitted with pneumococcal bacteremia caused by the same pathogen, *S. pneumoniae* strain SP225994. Antimicrobial susceptibility testing showed that the strain was highly resistant to levofloxacin. Both strains were identified as belonging to serotype 6B and resolved into sequence type 90 (3). 

A total of 70 ng/µL genomic DNA was extracted from the two samples using a commercial kit (Qiagen DNeasy kit). Adaptors were added to each library during preparation according to the TruSeq protocol (Illumina) to produce multiplexed paired-end libraries. The samples were run on a sequencer (Illumina MiSeq) at the Technion Genome Center, Haifa, Israel, generating 8,632,644 and 8,110,099 paired-end-reads for strains SP224896 and SP225994, respectively. Reads from the Illumina MiSeq platform were trimmed for low-quality regions and adaptor sequences using Trim_Galore (http://www.bioinformatics.babraham.ac.uk/projects/trim_galore). For assembly of the reads, further improvement of the read quality was achieved using Trimmomatic version 0.32 (4). The quality of the reads was verified prior to and after quality trimming with FastQC version 0.11.2 (http://www.bioinformatics.babraham.ac.uk/projects/fastqc). The SPAdes version 3.1.1 assembler was used to assemble the Illumina reads (5), and Prokka version 1.11 (6) was used for gene annotation with the *S. pneumoniae* R6 genome (accession no. GCF_000007045.1) used as the reference genome.

The draft genome sequences consisted of 237 contigs totaling 2,463,761 bp and 2,473,562 bp in length for strains SP224896 and SP225994, respectively, with 2,412 coding sequences. A total of 432 new variants were identified in the draft genome of strain SP225994 (accession no. LRSN00000000) that were absent from the draft genome of strain SP224896 (prior to levofloxacin therapy) (accession no. LRTZ00000000); this included 88 point mutations in the *gyrA* gene coding for DNA gyrase.

### Nucleotide sequence accession numbers.

This whole-genome shotgun project has been deposited at DDBJ/EMBL/GenBank under the accession numbers LRSN00000000 and LRTZ00000000. The versions described in this paper are the first versions, LRSN01000000 and LRTZ01000000.
